# How interest groups influence public opinion: Arguments matter more than the sources

**DOI:** 10.1111/1475-6765.12298

**Published:** 2018-07-13

**Authors:** ANDREAS DÜR

**Affiliations:** ^1^ Department of Polticial Science University of Salzburg Austria

**Keywords:** climate change agreement, interest groups, public opinion, source cues, Transatlantic Trade and Investment Partnership (TTIP)

## Abstract

Through what mechanism do interest groups shape public opinion on concrete policies? In this article, three hypotheses are proposed that distinguish between the effect of the arguments conveyed by interest groups and the effect of interest groups as source cues. Two survey experiments on the proposed Transatlantic Trade and Investment Partnership (TIPP) and the 2015 Paris Agreement on climate change allow the testing of these hypotheses. The resulting evidence from several countries shows that, with respect to interest groups’ attempts at shaping public opinion, arguments matter more than their sources. This is so even when accounting for people's trust in the interest groups that serve as source cues and for people's level of information about a policy. The finding that interest groups affect public opinion via arguments rather than as source cues has implications for the literature on elite influence on public opinion and the normative evaluation of interest group activities.

## Introduction

Much evidence suggests that interest groups not only respond to, but also try to sway public opinion. As early as the 1950s, Truman (1951: 213) concluded that interest groups engage in ‘more or less continuing efforts to guide and control’ public attitudes. He even postulated that ‘almost invariably one of the first results of the formal organization of an interest group is its embarking upon a program of propaganda, though rarely so labelled, designed to affect opinions concerning the interests and claims of the new group’. Nearly half a century later, Kollman (1998) found that for 56 per cent of interest groups, ‘the public’ was the primary target of their campaigns. Many studies of interest group strategies come to similar conclusions (Schlozman & Tierney 1986; Baumgartner et al. 2009; Dür & Mateo 2013). This evidence begs two questions: Do these activities by interest groups actually change individual attitudes with respect to specific policies? And if so, how does this effect come about?

Drawing on the literature on how political parties affect public opinion (e.g., Cohen 2003; Bullock 2011; Druckman et al. 2013; Leeper & Slothuus 2014), I develop three hypotheses that distinguish between the effect of the arguments conveyed by interest groups and the effect of interest groups as source cues. By ‘argument’ I mean a statement given in support of a specific idea. This definition subsumes, but is broader than, the definition of a ‘frame’, which is a statement that has the potential to change the weights that recipients give to different beliefs. Arguments can change both the weights attached to beliefs and the beliefs themselves. The literature on framing still is relevant for the argument that I present below. By ‘source cue’ I mean information on the source of an argument that potentially allows recipients to infer something about the argument itself.

I test the theoretical expectations using original data from two survey experiments, one on the Transatlantic Trade and Investment Partnership (TTIP) and the other on the Paris Agreement on climate change. The results show that interest groups can indeed affect public opinion on some issues. They mainly do so via the arguments they convey, whereas interest groups matter little as source cues. The relative absence of an interest group source cue effect even holds for people that trust the interest groups. Moreover, whereas the effect of interest groups’ arguments is particularly pronounced for people with relatively little information about a policy, the amount of information that citizens possess does not condition the effect of interest group source cues.

These findings make a key contribution to a literature that studies the impact of interest group activities on public attitudes. Whereas much research assumes such an impact (e.g., Schattschneider 1960; Keck & Sikkink 1998; Berry 1999; Weakliem 2003; Kriesi et al. 2007; Waterhouse 2013), studies that directly and systematically assess the effect of interest group campaigns on public opinion are relatively rare. In an early publication, Page et al. (1987) concluded that when interest groups push in one direction, public opinion more often than not moves in the other. A study of the effect of interest group campaigning on five ballot initiatives in California also came to the conclusion that interest groups can find it difficult to positively shape public opinion (Lupia 1994). In an experimental study, Nicholson (2011) found that support for a fictitious ballot proposal was the same when it was sponsored by the tobacco industry and when it was sponsored by a public health advocacy group. McEntire et al. (2015), finally, show how different types of frames that human rights organisations use matter for citizens’ attitudes towards human rights abuses.

Several case studies also assessed the impact of specific interest group campaigns on public opinion (Burstein 1985; Wlezien & Goggin 1993; Andsager 2000; McKnight & Hobbs 2013; Dür & Mateo 2014). While some of them find little evidence of an impact of these campaigns (e.g., Andsager 2000), others offer evidence of interest groups’ ability to push public opinion in the desired direction (McKnight & Hobbs 2013; Dür & Mateo 2014). A few studies also look at the impact of interest group endorsements on support for specific political candidates (Arceneaux & Kolodny 2009; Weber et al. 2012; Neddenriep & Nownes 2014). Most of them find that interest groups can influence vote choices. Weber et al. (2012) argue that this effect is conditional on the credibility of the source. Arceneaux and Kolodny (2009), however, show that a liberal interest group's endorsement of a Democratic candidate made self‐identified Republicans less likely to support that candidate.

This article adds to this literature by distinguishing between interest groups’ effects as source cues and via arguments. While a few earlier studies have also made this distinction, this article is the first that can empirically separate these two effects for the case of interest groups. Second, the empirical test of the argument relies on survey experiments. Some studies have used experiments to examine interest groups’ influence on support for political candidates, but so far experiments have hardly been used to study the impact of interest groups on attitudes towards concrete policies. Third, the experiments rely on real‐world examples that ensure exogenous variation in the amount of information that people have about a policy. Finally, whereas nearly all research so far has focused on the United States, this study relies on data from European countries with different systems of interest representation. It thus not only changes the institutional context, but allows for a test of cross‐national differences.

## Interest group influence on public opinion

An interest group that strongly favours a specific policy, but is unable to convince decision makers of the policy's merits, may try to have an indirect impact on decision making by mobilising and shaping public opinion. It can do so by using both new and traditional media, sending leaflets to households or holding a rally. Greenpeace, for example, has used campaign posters, launched a campaign website and organised an online petition in its attempts to stop the negotiations for a TTIP. Does this effort actually change public opinion, in the sense of making people more or less supportive of the policy? If so, through which mechanism does such outside lobbying matter for public opinion?

Much research suggests that political elites can indeed shape individual attitudes (Zaller 1992; Chong & Druckman 2011). This literature builds on the premise that most individuals are ‘awash in ignorance’ of politics (Kinder 1998: 784; see also Zaller 1992). Citizens’ lack of information may make it possible for political elites to shape public opinion via issue frames (also called ‘emphasis frames’) that stress a specific interpretation of an event (Chong & Druckman 2007; Nelson 2011). Following Druckman (2004: 672), issue frames ‘focus on qualitatively different yet potentially relevant considerations’ of an issue.^1^ For example, a smoking ban can be framed as a public health issue or as government interference with personal lifestyles. The impact of frames can best be understood when conceiving of attitudes as the sum of a set of beliefs or evaluations and weights for each belief (Nelson & Oxley 1999). The emphasis put on specific issue frames may change the relative weights of the evaluations, by making one or several evaluations more accessible and applicable to an issue. The result is a change in overall attitude. A framing effect hence implies that ‘a speaker's emphasis on a subset of potentially relevant considerations causes individuals to focus on these considerations when constructing their opinions’ (Druckman & Nelson 2003: 730). In addition to this framing effect, statements by political elites may also have an impact on attitudes by changing the beliefs themselves. This is then a persuasion effect.

Because there is little reason to believe that the arguments conveyed by interest groups are qualitatively different from the arguments transmitted by other political elites, the finding of framing and persuasion effects should also apply to interest groups. Clearly, however, not all arguments transport the same amount of information. Much research shows that ‘frame strength’ matters for the effect of frames on individual attitudes (Chaiken & Maheswaran 1994; Druckman et al. 2013). The issue frames that transmit concrete information (‘strong frames’) should matter more than vague ones (‘weak frames’). The same can be expected for arguments more broadly. I thus hypothesise:

*H1*: Strong pro/con arguments used by interest groups increase/decrease public support for a policy.

Another way for citizens to make political judgments in the absence of much information is to rely on source cues (Druckman 2001; Cohen 2003; Arceneaux & Kolodny 2009). A cue is a piece of information that people use to infer information that they do not possess. When relying on source cues, individuals transfer information about political actors to policies. For example, people that consider a specific political actor highly credible may react to an endorsement of a specific policy by that actor with support for the policy. By contrast, people that consider that actor not credible may react to the same endorsement by becoming more sceptical of the policy. In some cases, a source cue may trump the information contained in a message. As put by McGuire (1969: 198): ‘When the purported source is clearly positively or negatively valenced, he [the message receiver] uses this information as a cue to accept or reject the message's conclusions without really absorbing the arguments used.’

Research on the impact of political parties on public opinion debates the relative importance of issue frames and source cues in shaping individual attitudes (Cohen 2003; Bullock 2011; Druckman et al. 2013; Leeper & Slothuus 2014). Whereas some studies put emphasis on the effect of issue frames (Bullock 2011), others assign a greater role to party source cues (Cohen 2003). To resolve this controversy, some authors try to uncover the factors that make either issue frames or source cues more important. Slothuus and De Vreese (2010), for example, suggest that more politically aware people react more strongly to party source cues relative to issue frames than less politically aware people. For people willing to invest effort in forming their attitudes, by contrast, source cues should matter less than other information they possess or receive (Leeper & Slothuus 2014). Strong party polarisation on an issue also increases the relative importance of party source cues (Druckman et al. 2013).

This discussion can be translated to the case of interest groups as source cues. Most interest groups are not the ‘clearly positively or negatively valenced’ sources that McGuire (1969: 198) refers to as ‘cues’. Weber et al. (2012: 567), however, argue that some ‘interest groups will be persuasive precisely because they lack reputations and their political motives are unclear’. Moreover, some interest groups (such as Greenpeace) are very well known even to people with low political awareness. For other groups, their names clearly indicate what they stand for (e.g., Climate Justice Now!). From the information that Greenpeace opposes a policy, individuals may infer that this policy is likely to harm the environment as they know that Greenpeace is an environmental nongovernmental organisation (NGO). Interest group cues may also tell citizens who the likely winners and losers of a policy are, which in turn may allow them to adopt a position on that policy.

In fact, a few earlier studies found a cueing effect for interest groups (Lupia 1994; Arceneaux & Kolodny 2009; Neddenriep & Nownes 2014). These studies, however, did not distinguish between the impact of the source cue and the argument transmitted by groups. In fact, Arceneaux and Kolodny (2009: 759) explicitly state that their study design does not allow them to distinguish between the effect of the source cue and the message transmitted by the source. Similarly, in their study of the effect of interest group endorsements of presidential candidates, Neddenriep and Nownes (2014) confronted their subjects with interest group endorsements that also contained information about the candidates’ stance on abortion.

To capture the source cueing effect, however, it is not only important to disentangle the effects of cues and of arguments. A source may have a positive effect on some people (those that trust the source) and a negative effect on others (those that distrust the source). A large literature suggests that source credibility and likability matter for the persuasiveness of a message (e.g., McGuire 1969: 182–194; Petty & Cacioppo 1986: 153–156; Eagly & Chaiken 1993: 247–250; Lupia 1994; Pornpitakpan 2004). People that trust an interest group may align their attitudes with the positions advocated by that group. By contrast, people that distrust the interest group may shift their attitudes further away from the positions advocated by that group. In the aggregate, these two effects may offset each other. Summarising this discussion about interest groups as source cues, I thus hypothesise:

*H2*: An interest group source cue enhances public support for a policy among people that trust the interest group, but lowers support for the policy among people that do not trust the group.

The importance of both arguments and interest group source cues should be particularly large for people with little information about a policy. People that possess much information about a policy will neither need an argument nor a cue to establish their attitudes. Any information contained in a message or a source cue most likely is already part of their attitudes. Even if the information transmitted via an argument or an interest group source cue is new for people with high issue‐relevant knowledge, they are likely to have greater confidence in their attitudes, and thus are less likely to change them in response to an argument or a cue than people with little information. People with little information about a policy, by contrast, may use information contained in a message to adjust their attitudes. This is most likely for arguments that contain concrete information – that is, strong arguments. Citizens with little information about a policy may also want to use source cues to form their attitudes (e.g., Wood & Kallgren 1988). In the form of a hypothesis:

*H3*: The effect of arguments and interest group source cues is particularly large for people with little information about a policy.

I rely on two experiments embedded in public opinion surveys to test these hypotheses. Such experiments have become an important tool for the social sciences (see Mutz 2011), but they have hardly been used to investigate the impact of interest groups on public attitudes towards specific policies. In survey experiments, one or several elements of the questionnaire used are systematically varied across respondents (e.g., some respondents are told that an interest group supports a policy, whereas others do not get that information, before a question on whether the respondents approve or disapprove of the policy), with random assignment of respondents into treatment and control groups. Variation between treatment and control groups, and across different treatment groups, can be attributed to variation in the treatment that respondents received. While clearly survey experiments are no panacea to all research design problems, they offer a unique opportunity to tackle some of the problems hampering observational research on the impact of interest groups on public opinion. Survey experiments combine the advantages of the traditional experimental set‐up (internally valid causal inference because of random assignment) with the added strengths that participants can be representative for a given population and that sample sizes tend to be large. The choice of real‐world examples, including interest groups that are really active on these cases and arguments that are prominent in these debates, should partly alleviate concerns about the external validity of the findings.

## The TTIP experiment

### Methodology

The topic that I chose for the first survey experiment is the proposed TTIP between the European Union (EU) and the United States. The TTIP negotiations started in 2013 with the aim of liberalising trade between, and protecting foreign direct investments in, the EU and the United States. Especially in Europe, early public opposition to these negotiations emerged. Several prominent NGOs, including Greenpeace and Friends of the Earth, were active in encouraging this opposition. While this public opposition did not lead to an end of the negotiations, it forced the EU to change its approach. As of mid‐2017, the negotiations are on hold.

The choice of a real‐world example such as TTIP has the advantage that it comes with exogenous variation in the amount of information that people possess. I need such variation to test *H3*. A real‐world example also increases the external validity of the experiment. Furthermore, the selection of this topic had the advantage that it allowed me to carry out my experiment in several countries (for the country selection, see below) as it is one to which respondents across many countries can relate.

Respondents in the control group were simply asked about their attitude towards this trade agreement (see Table A‐1 in the Online Supporting Information for the wording of the experiment in the United Kingdom). They could respond on a seven‐point scale from ‘strongly oppose’ to ‘strongly support’ (with the possibility to opt for ‘don't know’). Across the 12 treatment groups, I varied both the source cue (no source cue when respondents were just told about supporters or opponents, and source cues when either ‘business associations’ or specific interest groups were mentioned) and the argument (statements transmitting a weak and a strong argument for both the pro and the con sides of the debate). The exact source cues vary by country: in each country, I used the names of the most prominent peak national business association (e.g., the Confederation of British Industry for the United Kingdom) and of two NGOs: Friends of the Earth (in Germany BUND) and Greenpeace. I used well‐known groups as most people can only be expected to trust or distrust well‐known groups.^2^ Moreover, the choice of both business groups and citizen groups allows to see whether they differ in terms of their effect as source cues, as expected for example by Page et al. (1987).

The weak arguments were formulated to be as generic as possible (e.g., the agreement will benefit the economy), whereas the strong arguments contain precise information (e.g., it will lead to the creation of 150,000 new jobs). For the strong con argument, I used the topic of investor protection as this is key in the arguments by the agreements’ opponents.^3^ Given this set‐up, the difference in public opinion between the scenario with (without) interest group source cue and a weak (pro or con) argument and the scenario with (without) interest group source cue and a strong (pro or con) argument captures the effect of arguments. The difference in public opinion between the scenario with a weak (strong) pro (con) argument with interest group source cue and a weak (strong) pro (con) argument without interest group source cue captures the cueing effect.

The distinction between weak and strong arguments also allows me to assess whether the effect of arguments dominates the source cueing effect in the experiment. Such a domination would lead me to the wrong conclusion that there is no cueing effect. However, if source cues matter, their effect should be visible when they appear in combination with the weak arguments, even if they do not matter in combination with the strong arguments.

The survey was fielded by the polling company YouGov to its online panels in France, Germany and the United Kingdom between 18 February and 6 March 2015. These online panels are actively recruited by YouGov. A key advantage of online surveys relying on large panels is that they are cost‐efficient. At the same time, online panels may suffer from selection bias. To reduce this problem, quotas ensure the representativeness of the samples for the voting populations of the three countries with respect to gender, age and region.^4^ While selection bias may still persist, at a time when phone surveys struggle with low response rates this problem is not unique to online surveys.

For each country, the survey has between 2,160 and 2,388 valid responses, for a total N of 6,826 (see Table A‐3 in the Online Supporting Information).^5^ Of them, 753 are in the control group, 3,226 were given an argument that supports TTIP and 3,128 were given an argument that opposes TTIP. A total of 2,242 respondents received a business cue and 2,241 respondents received a citizen group cue. The various groups are very similar in terms of both demographics (gender, age group, university education) and ideology (left‐right placement). This indicates that the random assignment to groups was effective.

A manipulation check shows that the treatment worked as expected. After the experiment, I asked respondents to evaluate the positions of a series of actors on TTIP.^6^ Those that received the treatment with the named peak business association were considerably more likely to indicate that this organisation strongly supports TTIP (45 per cent in the treatment group with a specific business cue and a weak pro argument, and 42 per cent in the treatment group with a specific business cue and a strong pro argument, as compared to 31 per cent in the control group).^7^ Equally, those reading the treatments containing Friends of the Earth and Greenpeace were more likely to respond that these organisations strongly oppose TTIP (32 and 40 per cent in the two groups that received a Friends of the Earth treatment, as compared to 21 per cent in the control group for Friends of the Earth; and 33 and 47 per cent in the two groups that received a Greenpeace treatment, as compared to 23 per cent in the control group for Greenpeace). This evidence indicates that the treatment with both the source cues and the arguments was effective.

Having data from three different countries is a major asset of this study as it makes sure that the results are not driven by the idiosyncrasies of a specific country. I opted for these three countries because they differ in the system of interest representation, with France resembling the statist model, Germany the neocorporatist model and the United Kingdom the pluralist model. At the same time, the three countries are similar with respect to many other variables (geographic location, size, level of economic development and regime type). For the specific survey experiment with respect to TTIP, this case selection is also interesting because the three countries differ in the strength of an interest group campaign against TTIP. This campaign was particularly strong in Germany, followed by France, whereas the topic is of little public salience in the United Kingdom. These differences create cross‐country variation in terms of the information that respondents can be expected to have about the negotiations.

*H2* refers to people's trust in interest groups. To measure this variable, the survey contained a question (prior to the experiment) asking respondents to indicate, on an 11‐point scale (from ‘no trust at all’ to ‘complete trust’), how much they ‘personally trust each of the following institutions or organisations’. The list included a total of 14 institutions or organisations (which were presented in random order), among them the various interest groups used as source cues in the survey experiment (*Trust business*, *Trust business specific*, *Trust Friends of the Earth* and *Trust Greenpeace*). Trust in the two NGOs tends to be higher across all three countries than trust in business associations (see Figure A‐3 in the Online Supporting Information). Trust in the peak French business association, Mouvement des entreprises de France (MEDEF), is particularly low. Respondents that trust Greenpeace also tend to trust Friends of the Earth; and respondents that trust business associations in general also tend to trust the peak business association of their country. By contrast, respondents that trust business associations do not tend to trust Greenpeace or Friends of the Earth. This suggests that trust in these groups is a function of more general attitudes that people hold. This conclusion is substantiated by the fact that members in environmental organisations express considerably more trust in Friends of the Earth and Greenpeace than other people; and members of business or professional associations show greater trust in business associations.^8^

The experiment was preceded by a question asking respondents about how well informed they feel about the proposed trade agreement (with four response categories, from ‘not well informed at all’ to ‘very well informed’). This question allows me to measure the predictor mentioned in *H3* – namely previous level of information about a policy (*Information*). The data show that only a small minority sees itself as very well informed (see Figure A‐2 in the Online Supporting Information). At the same time, they reveal major differences in people's self‐perception of how much they know about TTIP across the three countries. Germans perceive themselves to be considerably better informed about this proposed trade agreement than British and French citizens. Given the strong interest group campaign about TTIP in Germany, this finding is not surprising. It suggests that the effect of the experimental treatment should be weakest in Germany because there the information contained in the treatment should already form part of the attitudes of many people. *Information* is positively correlated with respondents’ interest in politics and news consumption, but these correlations are relatively weak. Even regular consumers of news do not feel well informed at all about TTIP, despite considerable media attention to the issue at least in Germany. The self‐perception of information still seems to be a good measure of actual information as the respondents that saw themselves very well informed did much better in the knowledge questions included as manipulation checks (considering just those respondents in the control group, to avoid this check being influenced by the experiment). Those indicating that they are not well informed at all about TTIP were also much more likely to choose the ‘do not know’ response when asked about their approval of TTIP.^9^

In all models below, I add country dummies for Germany and the United Kingdom. The rationale for this is that public opinion with respect to TTIP varies considerably across the three countries in which the experiments were carried through (European Commission 2014). In Europe, Germans are, after Austrians, the most sceptical about this agreement, with a slight majority opposing a trade and investment agreement between the EU and the United States (39 to 41 per cent, according to the European Commission (2014: 202)). The French have a slightly more positive view of such an agreement (50 to 32 per cent), and the British are among the most supportive in the EU (65 to 19 per cent). This may reflect differences in general attitudes towards trade across the three countries. As there are some differences across countries in terms of the size of the various treatment groups (see Table A‐3 in the Online Supporting Information), controlling for country is necessary to reduce noise in the estimates of the treatment effects.

In robustness checks, I also add two demographic controls: age and gender. Younger citizens may react more strongly to source cues and arguments than older citizens as the latter tend to have more stable attitudes (Alwin & Krosnick 1991). Moreover, some studies have shown that it is easier to influence the attitudes of females than the attitudes of males (McGuire 1969: 251; Petty & Brinol 2010). *Age* is an ordinal variable with five values, which capture different age groups. *Female* captures the gender of the respondents. Keeping the number of covariates very low in these models is in line with the relevant methodological literature (Mutz 2011: 123–126). Table A‐2 in the Online Supporting Information contains summary statistics for the various variables.

### Empirical analysis

**Figure 1:**
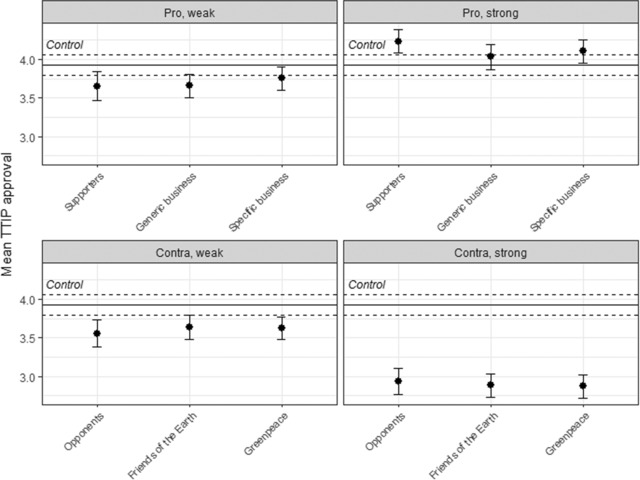
Attitudes towards TTIP. Notes: The solid horizontal line indicates the mean TTIP approval in the control group; the dashed horizontal lines indicate the 90 per cent confidence intervals for the control group. The whiskers show the 90 per cent confidence intervals for the treatment groups. Here and below I use 90 per cent confidence intervals to facilitate assessments of statistical significance: if two 90 per cent intervals do not overlap, the null hypothesis of no difference between two means can be rejected with at least a 95 per cent probability.

I start the empirical analysis by presenting descriptive evidence on the dependent variable. Figure 1 shows the mean responses to the question about TTIP approval by treatment group.^10^ As expected in *H1*, the figure shows a framing effect, which is particularly pronounced for the strong con argument. Surprisingly, the effect of the weak pro argument is to reduce TTIP approval (when combining the three groups that received different source cues, this effect is statistically significant at the 95 per cent level). By contrast, the source cues do not matter at all. In fact, the various treatment groups that received the same argument show very similar results. This is independent of whether respondents received a business cue (possibly indicating a narrow interest) or a citizen group cue (possibly indicating a public interest). Moreover, the absence of a source cueing effect is persistent across weak and strong arguments. Given that weak arguments transmit less information, one could have expected source cues to matter more in combination with them. Individual attitudes on TTIP thus can be influenced, but arguments clearly matter more than interest group source cues.

The pattern discerned in Figure 1 is stable across all three countries (see Figure A‐1 in the Online Supporting Information). Differences across groups with and without source cues, but exposed to the same argument, are minimal. The strength of the framing effect, however, varies by country, with the United Kingdom showing the strongest effect. In that country, the percentage of respondents that are in favour of TTIP drops from 48 per cent for the group treated with the strong pro argument to 14 per cent for the group treated with the strong con argument. The effect is weakest in Germany, where citizens – according to their self‐perception – are best informed about TTIP. There, the difference between these two treatment groups only is 14 percentage points.

For a more systematic test of *H1*, I ran an ordinal logistic regression model with *TTIP approval* as dependent variable, and dummy variables for each of the four arguments, four source cues (generic business, specific business, Friends of the Earth and Greenpeace) and country fixed‐effects as predictors.^11^ In this set‐up, the effect of the arguments is assessed relative to the control group, and the effect of the source cues relative to the group without source cue but with the same argument. In line with the descriptive analysis just shown, three of the four coefficients for the arguments are statistically significant (the exception being the weak pro argument).^12^ By contrast, none of the coefficients for the source cues is statistically significant. When interacting the source cues with the weak arguments to see whether the cues matter more in that context, none of the coefficients for the interaction terms is statistically significant. Adding *Age* and *Female* as demographic controls does not change these results. Neither of the coefficients for these two controls is statistically significant. Interacting the arguments with the left‐right placement of the respondents shows that the strong pro argument has a larger effect on respondents on the right; by contrast, the effect of the strong con argument is independent of ideology.

**Figure 2:**
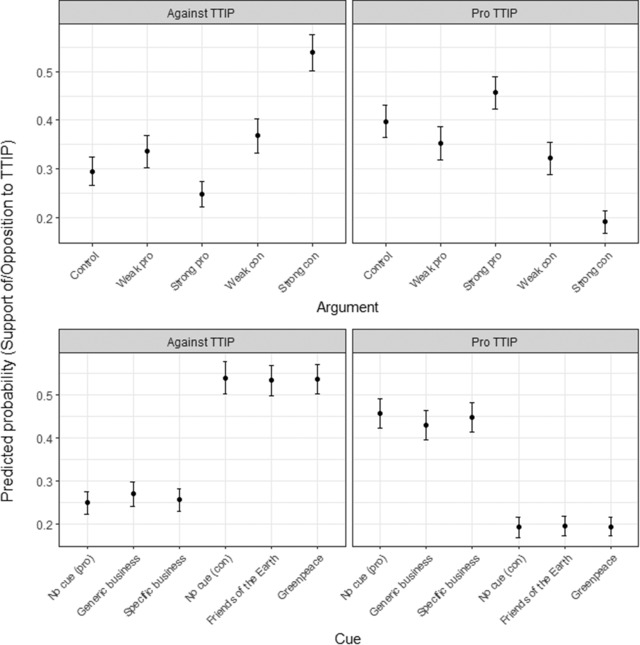
The predicted effects of the arguments and source cues. Notes: N = 4,903. I combined the three response categories indicating different degrees of opposition to (‘Against TTIP’) and support of TTIP (‘Pro TTIP’) to facilitate the presentation of the results. The whiskers show the 90 per cent confidence intervals.

In Figure 2, I show the predicted probabilities of supporting (from slightly to strongly) or opposing (also from slightly to strongly) TTIP based on the model without controls and interactions.^13^ Looking first at the effect of the arguments (the top two panes of the figure), the strong pro argument reduces opposition to and increases support for TTIP, but the substantive effect is modest. Whereas for the control group the probability of being in favour of TTIP (i.e., at least slightly support) is 40 per cent, the respective probability for the group exposed to the strong pro argument is 46 per cent. The effect of the strong con argument is much larger. For the group confronted with this argument, the probability of opposing TTIP increases to 54 per cent and the probability of being in favour of TTIP drops to 19 per cent. The difference between the strong pro and the strong con argument in terms of opposition to TTIP amounts to 29 percentage points. By contrast, I find little effect for the weak arguments. The effect of the weak pro argument is not statistically significant. The weak con argument slightly increases opposition to and reduces support for TTIP, but these effects are relatively small. These findings are robust when running the model separately for each of the three countries. Whereas the strength of the framing effect varies as discussed above, even in Germany the coefficient for the strong con argument is sizable and statistically significant. The evidence thus strongly supports *H1*.

The effect of the source cues is smaller than the effects of the arguments (see the bottom row of Figure 2). In fact, in no case is there a statistically significant difference in terms of TTIP approval between treatment groups that received different source cues but the same argument. This applies to both the weak and the strong arguments. The fact that I used high‐profile groups – the most prominent business associations and citizen groups in these countries – as source cues makes this finding particularly interesting. If there is any effect of interest group cues on individual attitudes, we should see it for these interest groups. Again, this effect is robust to running separate models by country. In none of these models, are the coefficients for the source cues statistically significant. The finding that in the aggregate, interest group source cues have at most a minor effect on attitudes is, thus, very robust.

This evidence, however, does not necessarily run counter to *H2*. There, the expectation was that the effect of interest group source cues should be conditional on trust. I test this specific argument by interacting the source cues with the various variables capturing trust in these specific actors. Otherwise, the set‐up of this model is the same as described above for the test of *H1*. The results for the arguments are similar as for the models described above (see Table A‐6 in the Online Supporting Information). Again, three of the four relevant coefficients are statistically significant. The main effects of the trust variables are in line with what one would expect: trust in business (both business associations generically and the specific peak business associations) goes hand‐in‐hand with a more positive attitude towards TTIP; trust in Friends of the Earth or Greenpeace is related to a more negative attitude towards TTIP.

**Figure 3:**
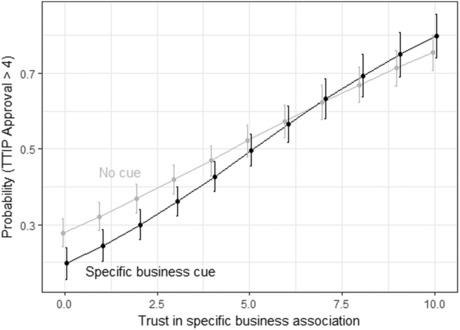
Illustrating the interaction effect: Trust × Specific business cue. Note: The whiskers show the 90 per cent confidence intervals.

More importantly, in this model the coefficient for the specific business cue is negative and statistically significant. Given that the model also contains interaction terms between the trust variables and the source cues, this negative coefficient means that for people that do not trust the specific business associations at all, mentioning them reduced their support for TTIP. In fact, the coefficient for the interaction between *Trust* and the specific business cue is also statistically significant. In Figure 3, I show this interaction graphically. As can be seen, the size of the effect is small. For people with very low trust in the specific business associations, mentioning their names has a small negative effect on TTIP approval. Since this is the only one of the four coefficients for the interaction terms that is statistically significant, and the substantive effect is small, overall the evidence does not offer much support for *H2*.^14^ Even when considering trust, interest group source cues do not have much impact on attitudes towards TTIP.^15^

In *H3*, I suggested that arguments and source cues are particularly important for people with little information about a policy. To test this hypothesis, I ran a model in which I interact the dummy variables for the various arguments and cues with *Information*, while adding country fixed‐effects as controls. In this model, three of the four coefficients for the arguments are statistically significant, while none of the coefficients for the source cues are statistically significant. More importantly, the coefficient for the interaction between the strong con argument and *Information* is statistically significant. The coefficient for the interaction with the strong pro argument is very close to statistically significant (p = 0.053). By contrast, none of the coefficients for the interaction terms between source cues and *Information* are statistically significant.^16^

**Figure 4:**
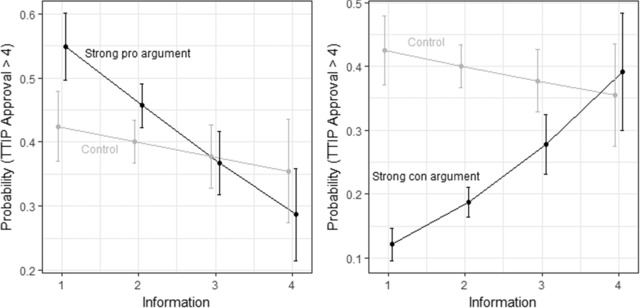
Illustrating the interaction effect: Information × Strong argument. Note: The whiskers show the 90 per cent confidence intervals.

In Figure 4, I show the substantive effects of the two interaction terms for the strong arguments graphically. Clearly, the strong con and strong pro arguments have opposite effects. The strong pro argument produced greater support for TTIP among respondents with little information about TTIP. The strong con argument had a major negative effect on respondents’ attitudes towards TTIP, with this negative effect only disappearing for respondents that consider themselves very well informed about TTIP. This is very much in line with the causal reasoning underlying *H3*. That the weak arguments, which transmit less information than the strong arguments, do not have the same effect also makes sense within this logic. These effects, however, contrast with the absence of a similar effect for the source cues. The evidence thus backs the part of *H3* related to arguments but not the part related to source cues.

## The experiment on the climate change agreement

I carried out a second experiment to respond to two potential criticisms of the TTIP experiment. For one, the TTIP experiment does not include a treatment in which respondents are provided only with a source cue without argument. It thus cannot exclude the possibility that source cues matter as long as they do not come with other information (although it indirectly tackled this issue by showing that the source cues have the same, close to zero effect, independent of the strength of the argument). The second experiment tackles this issue head‐on by having actors only take a pro or con position. Moreover, the TTIP experiment only covers ‘plausible’ combinations of source cues and arguments. For many respondents, it may not be very surprising that Greenpeace complains about a lack of transparency in the TTIP negotiations, or that a peak business association claims that TTIP will create jobs. The second experiment remedies this potential shortcoming by also including unexpected pairings betweeen source cues and arguments.

This second experiment focuses on environmental policy – concretely the climate change agreement reached in Paris in late 2015. This agreement, which has been signed by nearly all countries around the globe, aims to limit the increase in the global average temperature to less than 2 degrees Celsius above pre‐industrial levels. The negotiations leading up to this agreement were the focus of an interest group campaign, although the topic never garnered as much public attention as TTIP in Germany. By opting for a different topic, I can also use this experiment to see whether the virtual absence of a source cue effect evident in the TTIP experiment is specific to the trade policy realm.

The experiment was embedded in a public opinion poll fielded in Germany and Spain in June 2016. The online poll, which was carried out by respondi in Germany and Netquest in Spain, had 2,000 and 2,001 respondents, respectively. Quotas for age and gender ensure basic demographic representativeness of the samples. Respondents were randomly assigned to a control group (500 respondents per country) or one of six treatment groups (250 respondents for each of the six treatments in both countries). Respondents in the control group were asked: ‘At the end of 2015, 195 countries agreed on an international agreement on climate protection. Its aim is to limit the earth's temperature increase to 2 degrees Celsius. How do you view this agreement?’ The response scale had seven values and ranged from ‘strongly oppose the climate agreement’ to ‘strongly support it’.

In addition to the question wording above, respondents in the treatment group received information on an actor and its alleged position towards the climate agreement. The actors were Friends of the Earth (in their German and Spanish versions, respectively), large domestic companies (not further defined) and the peak business associations in the two countries (the Bundesverband der Deutschen Industrie and the Confederación Española de Organizaciones Empresariales, respectively). Each of these actors was said to take either a position for or against the climate agreement, before the question on how respondents view the agreement (the exact wording is contained in Table A‐8 in the Online Supporting Information).

**Figure 5:**
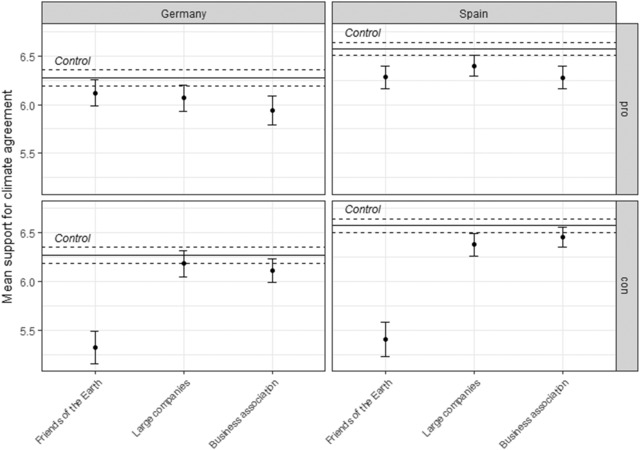
Support for the Paris climate agreement. Note: The whiskers show the 90 per cent confidence intervals.

Figure 5 shows the mean support for the climate agreement by country and treatment group, compared with support for this agreement in the control group. For the respondents that received the pro treatment, which actor's position the respondents learned about in the treatment hardly matters. Any effects that are statistically significant are substantively small. It may be argued that the high average support for the agreement in the control group introduces ceiling effects in the sense that further increasing this support is hardly possible. However, it is still interesting to observe that to the extent that the cues have any effect, they lower support for the agreement. For the respondents receiving the con treatment, the source did matter in one instance: when respondents learned that Friends of the Earth opposes the agreement, support for the agreement is considerably lower than in the control group as it is when large companies or the peak business associations are said to oppose the agreement. The effect is virtually the same in Germany and Spain, giving weight to this finding.

These findings thus add one caveat to the findings from the TTIP survey: while indeed interest group source cues seem to have only a small impact on individual attitudes, an interest group can matter as a source cue if it conveys counter‐intuitive information (see also Calvert 1985). The statement that an environmental NGO opposes an environmental agreement – which most respondents would assume to be good for the environment – evidently contains so much information that a considerable number of respondents adjusted their attitudes. Outside of an experimental setting, however, citizens will only rarely encounter such a counter‐intuitive interest group source cue.

For a test of *H2*, I ran an ordinal logistic regression model with support for the climate agreement as dependent variable, and interaction terms between trust (measured relying on the same question as used in the TTIP survey) and the various experimental treatments. Again, the results do not support *H2* (see Table A‐10 in the Online Supporting Information). Of the six coefficients for the interaction terms, only one (for the interaction trust in companies and the treatment in which companies speak out against the agreement) is statistically significant at the 95 per cent level. The negative coefficient for this interaction indicates that as trust in large companies increases, support for the climate agreement also increases. But it does so more rapidly for respondents that received a negative endorsement from big companies. This is a counterintuitive result that is not at all in line with the expectation summarised in *H2*. However, it is robust to estimating the model separately for Germany and Spain (see models 2 and 3 in Table A‐10 in the Online Supporting Information). Clearly, then, neither the TTIP nor the climate agreement experiment offer support for *H2*.

## Conclusion

Through which mechanism do interest groups affect public opinion? I have formulated and tested three hypotheses that distinguish between the effect of arguments and the effect of interest groups as source cues. The finding is that interest groups mainly matter for public opinion via the arguments that they convey. This effect is particularly large for people with little information about a policy. By contrast, interest groups only have a minor effect as source cues. Not even for people with high trust in an interest group does this basic finding change. The finding that arguments matter independent of the sources is particularly interesting given the commonly held view that people ‘only believe frames that come from sources they perceive to be credible’ (Druckman 2001: 1045). The only case where an interest group as source cue really mattered for individual attitudes was when this cue was counter‐intuitive. In reality this will happen rarely, as what happens frequently is no longer counter‐intuitive.

An explanation for the virtual absence of an interest group source cue effect may be that only few people like or dislike interest groups the way that people like or dislike political parties. Although the groups that I chose for the experiment are generally well known, they are still less prominent than most political parties with parliamentary representation. The increasing professionalisation of interest groups, with most members having no role in them besides providing financial support (Barakso & Schaffner 2008), makes identification even less probable. Whereas partisan identification has been equated with religious identification (Green et al. 2002: 4), no such interest group identification has been empirically established. In the absence of identification with a specific interest group, people are less likely to be driven to see the world through a specific ‘group lens’ (as compared to seeing the world through a partisan lens).

A possible caveat to the findings presented in this article is that they derive from survey experiments. Existing research shows that treatment effects shown in survey experiments tend to be weaker in the real world (Barabas & Jerit 2010). This is so because the treatment is often much more explicit in survey experiments than in the real world; citizens will often face conflicting claims in the real world; and people not interested in current affairs may not even be exposed to the arguments made by actors in the real world. While these are potentially valid criticisms, these effects should actually further strengthen the findings of the present article: if even in a survey experiment interest groups hardly matter as source cues, they should matter even less in the real world.

The article's findings contribute to the literature on how elites can shape public opinion. Research on the determinants of public opinion has made much progress over the last few years, but (while recognising that an effect of interest groups is plausible) has failed systematically to bring interest groups into the picture. The findings presented here indicate that this is a problematic omission, because the relationship between interest groups and public opinion differs from the relationship between political parties and public opinion. While much research shows that political parties matter as source cues at least for some citizens, interest groups have, at most, a minor effect. Future research on the elite‐public opinion linkage should thus not just focus on political parties or mass media, but take into account that different types of elites may have different effects on public opinion.

The article also speaks to a growing literature on interest groups’ lobbying strategies (e.g., Kollman 1998; Baumgartner et al. 2009; Dür & Mateo 2013). A key question that this literature deals with is why some groups focus more on outside lobbying – namely trying to influence public opinion – whereas other groups focus more on inside lobbying – namely directly affecting what decision makers do. The article contributes to this literature a better understanding of the conditions under which outside lobbying can be effective in swaying public opinion. Outside lobbying can be successful in influencing public attitudes towards an issue, but only if interest groups manage to convey strong arguments. The fact that in the TTIP experiment (and to some extent also in the climate agreement experiment) the con arguments had a greater impact than the pro arguments also suggests that campaigns that take a stance against an issue may be more successful than campaigns that favour a specific policy proposal. In fact, in the real‐world TTIP campaign, the con arguments have also got more traction than the pro arguments. These insights should help clarify when interest groups’ outside lobbying is actually aimed at influencing policy outcomes, and when the main purpose of that lobbying is to maintain or increase the group's membership. The article's evidence also suggests that the use of front groups by some (business) interests matters little in terms of directly influencing public opinion (although it may still help these interests to place their arguments in media and the public discourse).

Several recent studies also analyse how public opinion matters for interest groups’ chances to influence public policy (e.g., Burstein 2014). Some studies argue that interest groups can only exert influence if their demands do not run counter to public opinion (Smith 2000). Others maintain that lobbying trumps public opinion (Gilens & Page 2014). The evidence provided here about interest groups’ ability to shape public opinion suggests that the question whether public opinion or interest groups matter more for public policies needs to be reconsidered. The relationship between interest groups, public opinion and policy outcomes is even more complex than allowed for in these studies, which tend to treat public opinion as fixed.

The finding that interest groups matter more via the arguments they convey than as source cues also has important normative implications. Two opposing views exist with respect to the normative evaluation of elite influence on public opinion. On the one hand, some authors warn of the perils of elite influence on public opinion (Kuklinski & Hurley 1994; Le Cheminant & Parrish 2011). If the masses are susceptible to strategic communication by elites (such as interest groups), lies, manipulation and misinformation may mislead the public. Moreover, if competing elites manage to influence public opinion, the latter may become unstable, not allowing for any coherent policy to emerge. On the other hand, public opinion responding to new information may be good news, because ‘citizens whose attitudes are held so rigidly that they seek only to reinforce their existing views’ are unlikely to be a good basis for democracy (Chong & Druckman 2012: 319). Citizens that fail to change their position, for example, because they exhibit a partisan resistance to new information, avoid uncomfortable truths, or are dogmatic, closed‐minded or politically intolerant, may create more problems for democracy than citizens that change their opinions in response to elite communication. Elite influence on public opinion then may equate ‘mutual education’ (Mansbridge 2003) rather than manipulation.

Both sides of this debate make valid points and it seems entirely plausible that interest group influence on public opinion can result in both ‘enlightenment’ (i.e., a better understanding) and ‘deception’ (i.e., a wrong understanding) (for these terms, see Lupia & McCubbins 1998: 8). On balance, the findings presented in this study support an optimistic reading. That public opinion responds more to the arguments conveyed by interest groups than interest group source cues means that people do not follow at least this type of political elite ‘rather blindly’ (Lenz 2012: 3). Instead, they evaluate the contents of messages. Moreover, the framing effect is driven by respondents with little information about a policy, and thus the individuals most in need of information to make up their minds. Clearly, then, people try to make informed decisions. Public opinion thus reacts to interest groups’ outside lobbying largely in line with how normative political theory states it should. The relevant questions for a normative evaluation of interest group influence on public opinion then are which interest groups manage to convey their arguments to the public and how sincere the information is that interest groups transmit to the public.

## Supporting information


**Table A‐1: Wording of the TTIP survey experiment (British case)**
**Table A‐2: Summary statistics**
**Table A‐3: Number of valid responses by treatment and country**
**Table A‐4: Regression results (Hypothesis 1)**
**Table A‐5: Regression results (Hypothesis 1), contd**.
**Table A‐6: Regression results (Hypothesis 2)**
**Table A‐7: Regression results (Hypothesis 3)**
**Table A‐8: Wording of the climate agreement survey experiment (translated to English)**
**Table A‐9: Summary statistics (Climate change agreement)**
**Table A‐10: Regression results**
**Figure A‐1: Attitudes towards TTIP (by country)**
**Figure A‐2: Information about TTIP**
**Figure A‐3: Trust in interest groups**

## Notes

1 By contrast, so‐called ‘equivalence frames’ convey the same information in ‘logically equivalent ways’ (Druckman 2004: 672).

2 Responses to a question on trust in different actors and institutions confirm the assumption that these interest groups are well‐known. For example, only 6 per cent of German respondents ticked the ‘don't know’ option when asked to state their trust in Greenpeace, which is comparable to the 4 per cent that did so for the German government or the 7 per cent that did so for the United Nations. Across the three countries and various actors, there are only two exceptions to this: 28 per cent of British respondents opted for ‘don't know’ when asked to state their trust in the Confederation of British Industry and 30 per cent of French respondents did so for Friends of the Earth.

3 There are also a few country‐specific debates about TTIP, such as the effect of this potential agreement on health services in the United Kingdom, but they would not have worked well in the other countries.

4 Only respondents eligible to vote in the respective country were allowed to participate.

5 Before arriving at this number of valid responses, I dropped 315 respondents who spent less than four minutes on filling in the questionnaire. The median duration across all respondents was 9.9 minutes. Respondents who invested less than four minutes most likely could not read all the questions and definitely did not optimise responses. In fact, their answers to a set of knowledge questions, where they fared much worse than the other respondents, suggest that their response behaviour was arbitrary. Male and female respondents are equally likely to be ‘speeders’; but most of the speeders fall into the 25–44 age group. Greszki et al. (2015) show that excluding speeders reduces noise in the data.

6 See the Online Supporting Information for the exact wording of this and the other questions referred to in the main text.

7 Two‐sample tests for equality of proportions show that these and the following differences between proportions are statistically significant at *p* < 0.01.

8 For this test, I use data from the following question contained in the survey: ‘Are you a member of or active in any of the following groups or organisations?’ Respondents received a list of 11 different types of groups or organisations.

9 Again only for the control group: 49 per cent of those that indicated that they are not well informed at all about TTIP then picked the ‘do not know’ response, compared to 14 per cent for all others.

10 To calculate these means, I assumed that the seven‐point ordinal response scale is continuous (i.e., that I am dealing with equal interval data).

11 Brant tests show that the proportional odds assumption holds in these models.

12 Regression tables for this and the following models are available in the Online Supporting Information.

13 These probabilities are calculated for citizens in France. The substantive effect, however, is independent of the country that is used in the calculation.

14 Running the model separately by country shows that the interaction between *Trust* and the Greenpeace cue is statistically significant for the United Kingdom and Germany (see Table A‐6 in the Online Supporting Information). Again, however, no other coefficient for the interaction terms is statistically significant. What is more, in the case of Germany, the prediced effect is the opposite of the one expected in *H2*. The country‐level models thus also do not support *H2*.

15 I also ran this model with membership in an environmental association or a business/professional association instead of the trust variables, without this changing the substantive result: none of the coefficients for the interaction terms comes even close to statistical significance (whereas the direct effects of the membership variables are highly statistically significant).

16 When running the model separately by country, the coefficient for the interaction between *Information* and the Greenpeace cue is statistically significant for France and Germany (see Table A‐7 in the Online Supporting Information). The substantive effects of the two interactions, however, are tiny.
