# Supplementary material for: How interest groups influence public opinion: Arguments matter more than the sources
Source: Eur J Polit Res. 2018 Jul 13;58(2):514–35. doi: 10.1111/1475-6765.12298 (PMC6487962; doi:10.1111/1475-6765.12298)
Supplement: Supplementary file 1 — Table A‐1: Wording of the TTIP survey experiment (British case) Table A‐2: Summary statistics Table A‐3: Number of valid responses by treatment and country Table A‐4: Regression results (Hypothesis 1) Table A‐5: Regression results (Hypothesis 1), contd. Table A‐6: Regression results (Hypothesis 2) Table A‐7: Regression results (Hypothesis 3) Table A‐8: Wording of the climate agreement survey experiment (translated to English) Table A‐9: Summary statistics (Climate change agreement) Table A‐10: Regression results Figure A‐1: Attitudes towards TTIP (by country) Figure A‐2: Information about TTIP Figure A‐3: Trust in interest groups [file EJPR-58-514-s004.pdf]

Supplementary Information

TTIP survey experiment

Table A-1: Wording of the TTIP survey experiment (British case)

1	Control group	How do you view this planned trade agreement?
2	No cue, weak pro argument	Supporters argue that this planned trade agreement will benefit the British economy. How do you view this agreement?
3	Generic business cue, weak pro argument	Business associations argue that this planned trade agreement will benefit the British economy. How do you view this agreement?
4	Specific business cue, weak pro argument	The employers' association Confederation of British Industry [France: Mouvement des entreprises de France (Medef); Germany: Bundesverband der Deutschen Industrie] argues that this planned trade agreement will benefit the British economy. How do you view this agreement?
5	No cue, strong pro argument	Supporters argue that 150,000 new jobs would be created in Great Britain as a result of this planned trade agreement. How do you view this agreement?
6	Generic business cue, strong pro argument	Business associations argue that 150,000 new jobs would be created in Great Britain as a result of this planned trade agreement. How do you view this agreement?
7	Specific business cue, strong pro argument	The employers' association Confederation of British Industry [France: Mouvement des entreprises de France (Medef); Germany: Bundesverband der Deutschen Industrie] argues that 150,000 new jobs would be created in Great Britain as a result of this planned trade agreement. How do you view this agreement?
8	No cue, weak con argument	Opponents complain about the negotiations for this planned trade agreement taking place behind closed doors. How do you view this agreement?
9	Friends of the Earth cue, weak con argument	The environmental organization Friends of the Earth [Germany: Bund für Umwelt und Naturschutz Deutschland (BUND)] complains about the negotiations for this planned trade agreement taking place behind closed doors. How do you view this agreement?
10	Greenpeace cue, weak con argument	The environmental organization Greenpeace complains about the negotiations for this planned trade agreement taking place behind closed doors. How do you view this agreement?
11	No cue, strong con argument	Opponents warn that this planned trade agreement would allow foreign companies to sue Great Britain in private tribunals for billions in compensation. How do you view this agreement?
12	Friends of the Earth cue, strong con argument	The environmental organization Friends of the Earth [Germany: Bund für Umwelt und Naturschutz Deutschland (BUND)] warns that this planned trade agreement would allow foreign companies to sue Great Britain in private tribunals for billions in compensation. How do you view this agreement?
13	Greenpeace cue, strong con argument	The environmental organization Greenpeace warns that this planned trade agreement would allow foreign companies to sue Great Britain in private tribunals for billions in compensation. How do you view this agreement?

Exact wording of questions in the TTIP survey

1.) How much do you personally trust each of the following institutions or organizations? (scale from 0-10)

In randomized order:

- British Government
- Political parties
- House of Commons
- European Union
- United Nations
- Media
- Business associations
- Nongovernmental organizations (NGOs)
- Trade unions
- Amnesty International
- Confederation of British Industry
- Friends of the Earth
- Greenpeace
- Big companies

8.) The European Union and the United States are currently negotiating a trade agreement, called Transatlantic Trade and Investment Partnership or TTIP, that should facilitate trade between the two entities. How well informed do you feel about this planned agreement? (Not well informed at all, Not very well informed, Fairly well informed, Very well informed)

9.) Survey experiment (see Table A-1 above)

11.) In your opinion, to what extent do the following actors support or oppose the planned trade agreement between the European Union and the United States? (5-point scale from strongly opposes TTIP to strongly supports TTIP)

In randomized order:

- British Government
- Confederation of British Industry
- Conservative Party
- Friends of the Earth
- Greenpeace
- Labour Party
- National Farmers' Union
- Trades Union Congress

Attitudes towards TTIP (by country)

Figure A-1: Attitudes towards TTIP (by country)

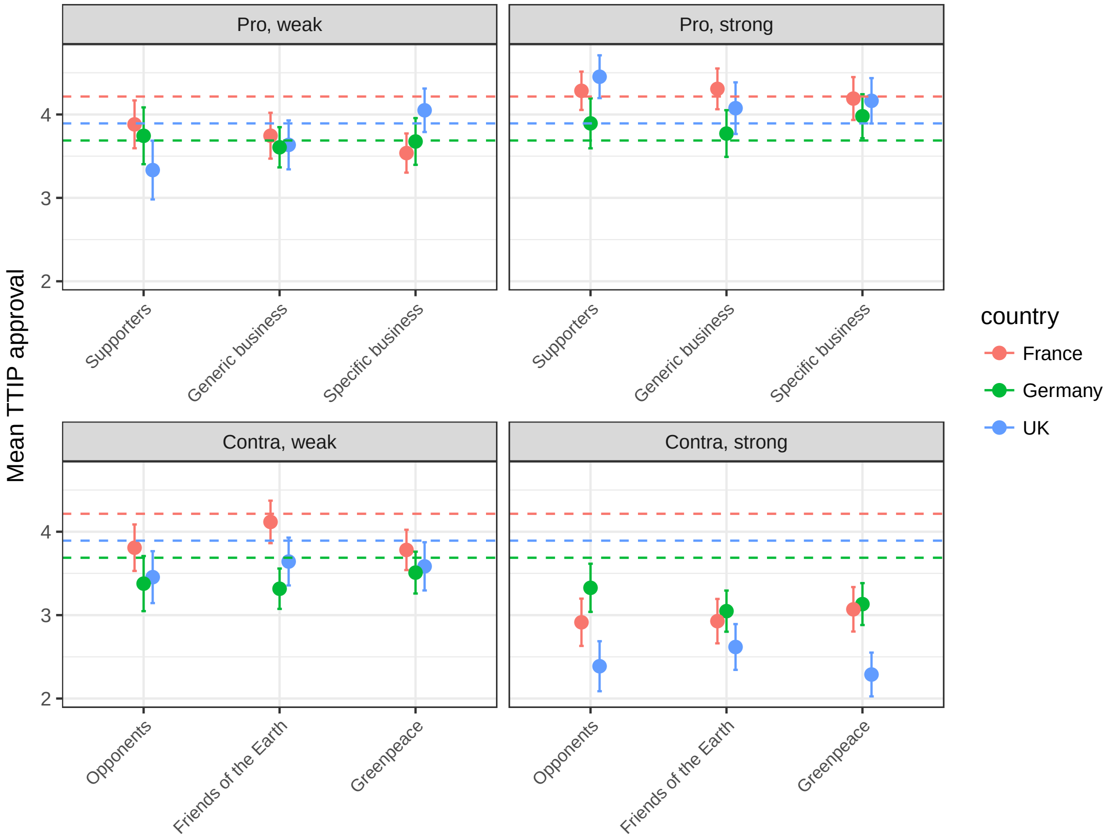

Note: The dotted horizontal lines indicate the means for the control group (by country).

Predictors (TTIP experiment)

Figure A-2: Information about TTIP

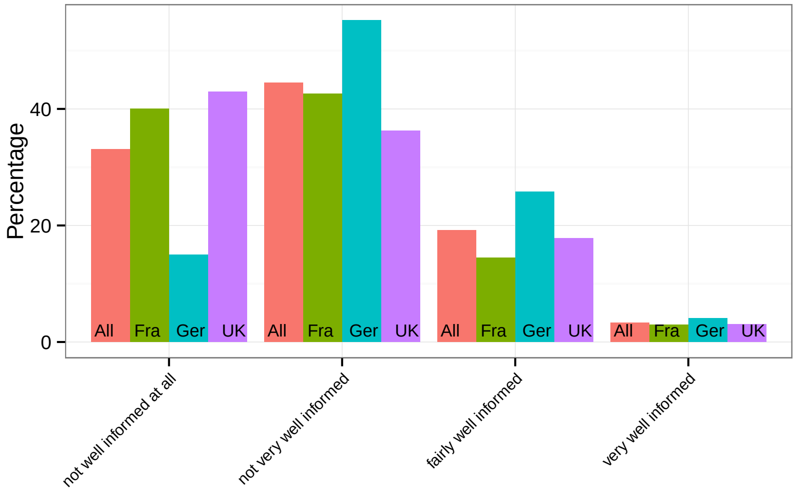

Figure A-3: Trust in interest groups

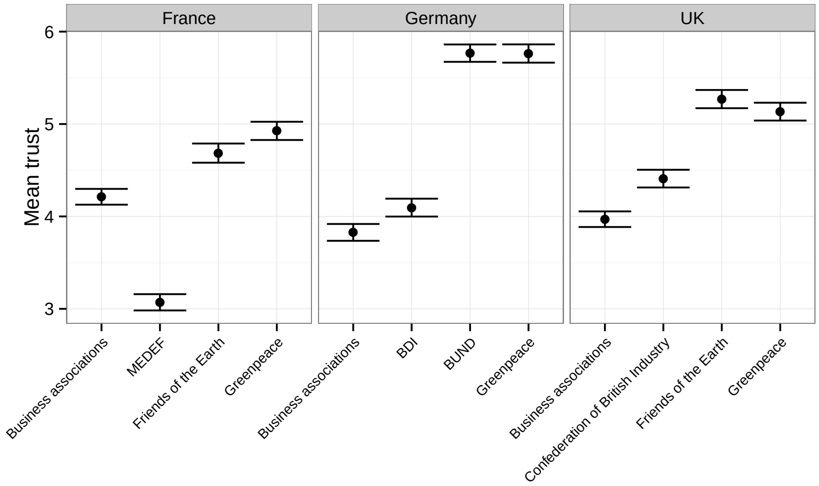

Note: The whiskers indicate the 90% confidence intervals.

Summary statistics (TTIP experiment)

Table A-2: Summary statistics

	N	Mean	St. Dev.	Min	Median	Max
TTIP approval	4,903	3.63	1.90	1	4	7
Trust business	5,895	4.01	2.36	0	4	10
Trust business specific	5,613	3.81	2.53	0	4	10
Trust Friends of the Earth	5,599	5.27	2.63	0	5	10
Trust Greenpeace	6,244	5.27	2.75	0	5	10
Information	6,826	0.93	0.81	0	1	3
Germany	6,826	0.32	0.47	0	0	1
United Kingdom	6,826	0.33	0.47	0	0	1
Age	6,826	3.67	1.34	1	4	5
Female	6,826	0.54	0.50	0	1	1

Number of valid responses by treatment and country (TTIP experiment)

Table A-3: Number of valid responses by treatment and country

**Groups**	**France**	**Germany**	**United Kingdom**	**Total**
Control	272	238	243	753
Generic pro, weak	138	116	162	416
Business pro, weak	167	183	173	523
Specific pro, weak	236	163	246	645
Generic pro, strong	202	142	187	531
Business pro, strong	165	165	167	497
Specific pro, strong	143	169	178	490
Generic contra, weak	148	115	156	419
Friends of the Earth contra, weak	157	187	169	513
Greenpeace contra, weak	197	187	160	544
Generic contra, strong	168	140	118	426
Friends of the Earth contra, strong	198	175	160	533
Greenpeace contra, strong	197	180	159	536
Total	2,388	2,160	2,278	6,826

Regression tables

Table A-4: Regression results (Hypothesis 1)

	**Model 1**	**Model 2**	**Model 3 (UK)**	**Model 4 (France)**	**Model 5 (Germany)**
Argument pro, weak	−0.190	−0.189	−0.182	−0.460	0.009
	(0.112)	(0.112)	(0.200)	(0.193)	(0.191)
Argument pro, strong	0.239	0.240	0.340	0.107	0.223
	(0.106)	(0.106)	(0.188)	(0.181)	(0.185)
Argument con, weak	−0.329	−0.333	−0.388	−0.468	−0.172
	(0.113)	(0.113)	(0.204)	(0.196)	(0.191)
Argument con, strong	−1.026	−1.030	−1.462	−1.472	−0.407
	(0.113)	(0.113)	(0.212)	(0.197)	(0.185)
Generic business cue	−0.109	−0.110	−0.105	−0.088	−0.113
	(0.095)	(0.095)	(0.167)	(0.167)	(0.162)
Specific business cue	−0.037	−0.039	0.120	−0.246	0.001
	(0.093)	(0.093)	(0.160)	(0.163)	(0.163)
Friends of the Earth cue	0.026	0.028	0.205	0.157	−0.177
	(0.098)	(0.098)	(0.180)	(0.175)	(0.160)
Greenpeace cue	0.011	0.009	−0.0003	0.109	−0.050
	(0.098)	(0.098)	(0.181)	(0.170)	(0.161)
Germany	−0.161	−0.162			
	(0.061)	(0.061)			
United Kingdom	−0.148	−0.150			
	(0.063)	(0.064)			
Age		−0.019			
		(0.019)			
Female		0.046			
		(0.051)			
N	4903	4903	1511	1603	1789
Log Likelihood	-8642.767	-8641.778	-2665.415	-2676.157	-3221.551

Note: Ordinal regression coefficients with standard errors in parentheses.

Table A-5: Regression results (Hypothesis 1), contd.

	Model 6	Model 7
Argument pro, weak	−0.280	−0.322
	(0.132)	(0.156)
Argument pro, strong	0.298	−0.289
	(0.115)	(0.268)
Argument con, weak	−0.372	−0.463
	(0.130)	(0.161)
Argument con, strong	−0.986	−1.274
	(0.129)	(0.285)
Generic business cue	−0.187	−0.203
	(0.128)	(0.132)
Specific business cue	−0.141	−0.166
	(0.126)	(0.129)
Friends of the Earth cue	−0.031	0.190
	(0.138)	(0.143)
Greenpeace cue	−0.046	−0.113
	(0.138)	(0.144)
Left right		0.004
		(0.042)
Germany	−0.160	−0.139
	(0.061)	(0.103)
United Kingdom	−0.148	−0.138
	(0.063)	(0.082)
Pro weak × Generic business	0.187	
	(0.191)	
Pro weak × Specific business	0.236	
	(0.187)	
Con weak × Friends of the Earth	0.115	
	(0.196)	
Con weak × Greenpeace	0.114	
	(0.195)	
Pro strong × Left right		0.161
		(0.076)
Con strong × Left right		−0.047
		(0.080)
N	4903	2399
Log Likelihood	-8641.691	-4189.122

NA

Note: Ordinal regression coefficients with standard errors in parentheses

Table A-6: Regression results (Hypothesis 2)

	**Model 8**	**Model 9**	**Model 10 (UK)**	**Model 11 (France)**	**Model 12 (Germany)**
Argument pro, weak	−0.076	−0.076	−0.148	−0.177	0.065
	(0.125)	(0.125)	(0.232)	(0.225)	(0.204)
Argument pro, strong	0.246	0.247	0.272	0.235	0.224
	(0.119)	(0.119)	(0.221)	(0.208)	(0.198)
Argument con, weak	−0.435	−0.437	−0.452	−0.675	−0.249
	(0.128)	(0.128)	(0.239)	(0.231)	(0.205)
Argument con, strong	−1.048	−1.051	−1.507	−1.389	−0.572
	(0.128)	(0.129)	(0.251)	(0.231)	(0.201)
Generic business cue	−0.049	−0.049	−0.056	−0.218	0.042
	(0.174)	(0.174)	(0.333)	(0.344)	(0.261)
Specific business cue	−0.449	−0.452	−0.169	−0.664	−0.367
	(0.169)	(0.169)	(0.348)	(0.279)	(0.284)
Friends of the Earth cue	0.118	0.117	0.091	0.479	−0.072
	(0.206)	(0.206)	(0.377)	(0.381)	(0.332)
Greenpeace cue	−0.175	−0.178	0.631	0.001	−0.848
	(0.197)	(0.197)	(0.369)	(0.346)	(0.322)
Trust business	0.189	0.190	0.163	0.127	0.233
	(0.019)	(0.019)	(0.038)	(0.034)	(0.032)
Trust business specific	0.208	0.207	0.253	0.218	0.170
	(0.018)	(0.018)	(0.037)	(0.029)	(0.031)
Trust Friends of the Earth	−0.079	−0.079	−0.119	−0.020	−0.088
	(0.018)	(0.018)	(0.041)	(0.032)	(0.028)
Trust Greenpeace	−0.052	−0.053	−0.021	−0.054	−0.057
	(0.017)	(0.017)	(0.039)	(0.028)	(0.025)
Trust business ×	−0.021	−0.021	−0.009	0.011	−0.045
Generic business cue	(0.035)	(0.035)	(0.069)	(0.067)	(0.052)
Trust business specific ×	0.070	0.070	0.025	0.068	0.078
Specific business cue	(0.032)	(0.032)	(0.062)	(0.059)	(0.051)
Trust FoE × FoE cue	−0.031	−0.030	−0.020	−0.054	−0.022
	(0.032)	(0.032)	(0.062)	(0.065)	(0.048)
Trust Greenpeace ×	0.028	0.029	−0.119	0.024	0.129
Greenpeace cue	(0.030)	(0.030)	(0.058)	(0.055)	(0.047)
Germany	−0.243	−0.242			
	(0.073)	(0.073)			
United Kingdom	−0.335	−0.333			
	(0.079)	(0.079)			
Age		−0.012			
		(0.023)			
Female		0.028			
		(0.058)			
N	3904	3904	1131	1180	1593
Log Likelihood	-6534.588	-6534.323	-1884.762	-1887.245	-2690.121

Note: Ordinal regression coefficients with standard errors in parentheses

Table A-7: Regression results (Hypothesis 3)

	**Model 13**	**Model 14**	**Model 15 (UK)**	**Model 16 (France)**	**Model 17 (Germany)**
Argument pro, weak	−0.285	−0.285	−0.180	−0.774	0.184
	(0.194)	(0.194)	(0.331)	(0.313)	(0.392)
Argument pro, strong	0.499	0.495	0.486	0.071	0.855
	(0.180)	(0.179)	(0.298)	(0.291)	(0.373)
Argument con, weak	−0.481	−0.483	−0.731	−1.030	0.628
	(0.194)	(0.194)	(0.331)	(0.311)	(0.396)
Argument con, strong	−1.679	−1.682	−2.342	−2.414	0.123
	(0.193)	(0.193)	(0.337)	(0.309)	(0.389)
Generic business cue	−0.007	−0.007	0.124	0.134	−0.353
	(0.162)	(0.162)	(0.266)	(0.266)	(0.340)
Specific business cue	−0.151	−0.152	0.021	−0.251	−0.266
	(0.159)	(0.159)	(0.260)	(0.257)	(0.340)
Friends of the Earth cue	−0.028	−0.025	−0.0004	0.250	−0.570
	(0.168)	(0.168)	(0.281)	(0.267)	(0.353)
Greenpeace cue	0.077	0.073	−0.121	0.746	−0.783
	(0.167)	(0.167)	(0.286)	(0.261)	(0.356)
Information	−0.098	−0.096	−0.668	0.045	0.475
	(0.100)	(0.100)	(0.170)	(0.171)	(0.182)
Information ×	0.077	0.077	0.018	0.327	−0.170
Argument pro, weak	(0.148)	(0.148)	(0.246)	(0.254)	(0.283)
Information ×	−0.271	−0.266	−0.235	0.028	−0.542
Argument pro, strong	(0.140)	(0.140)	(0.231)	(0.238)	(0.275)
Information ×	0.134	0.134	0.264	0.687	−0.674
Argument con, weak	(0.152)	(0.152)	(0.265)	(0.262)	(0.282)
Information ×	0.611	0.611	0.805	1.099	−0.462
Argument con, strong	(0.148)	(0.148)	(0.267)	(0.256)	(0.266)
Information ×	−0.081	−0.083	−0.220	−0.240	0.208
Generic business cue	(0.126)	(0.126)	(0.204)	(0.223)	(0.245)
Information ×	0.125	0.124	0.130	0.035	0.223
Specific business cue	(0.123)	(0.123)	(0.198)	(0.217)	(0.246)
Information ×	0.058	0.058	0.252	−0.198	0.313
Friends of the Earth cue	(0.132)	(0.132)	(0.237)	(0.227)	(0.241)
Information ×	−0.048	−0.046	0.111	−0.761	0.597
Greenpeace cue	(0.131)	(0.131)	(0.239)	(0.222)	(0.246)
Germany	−0.194	−0.195			
	(0.062)	(0.062)			
United Kingdom	−0.154	−0.155			
	(0.064)	(0.064)			
Age		−0.016			
		(0.019)			
Female		0.045			
		(0.051)			
N	4903	4903	1511	1603	1789
Log Likelihood	-8601.679	-8600.871	-2603.110	-2642.201	-3205.718

Note: Ordinal regression coefficients with standard errors in parentheses

Climate agreement survey experiment

Table A-8: Wording of the climate agreement survey experiment (translated to English)

1	Control group	At the end of 2015, 195 countries agreed on an international agreement on climate protection. Its aim is to limit the earth's temperature increase to 2 degrees Celsius. How do you view this agreement?
2	NGO pro	At the end of 2015, 195 countries agreed on an international agreement on climate protection. Its aim is to limit the earth's temperature increase to 2 degrees Celsius. Non-governmental organizations such as Friends of the Earth (BUND) [Amigos de la Tierra España] support this agreement. How do you view this agreement?
3	NGO con	At the end of 2015, 195 countries agreed on an international agreement on climate protection. Its aim is to limit the earth's temperature increase to 2 degrees Celsius. Non-governmental organizations such as Friends of the Earth (BUND) [Amigos de la Tierra España] oppose this agreement. How do you view this agreement?
4	Companies pro	At the end of 2015, 195 countries agreed on an international agreement on climate protection. Its aim is to limit the earth's temperature increase to 2 degrees Celsius. Large German [Spanish] companies support this agreement. How do you view this agreement?
5	Companies con	At the end of 2015, 195 countries agreed on an international agreement on climate protection. Its aim is to limit the earth's temperature increase to 2 degrees Celsius. Large German [Spanish] companies oppose this agreement. How do you view this agreement?
6	Business associations pro	At the end of 2015, 195 countries agreed on an international agreement on climate protection. Its aim is to limit the earth's temperature increase to 2 degrees Celsius. Business associations such as the Bundesverband der Deutschen Industrie [Confederación Española de Organizaciones Empresariales] support this agreement. How do you view this agreement?
7	Business associations con	At the end of 2015, 195 countries agreed on an international agreement on climate protection. Its aim is to limit the earth's temperature increase to 2 degrees Celsius. Business associations such as the Bundesverband der Deutschen Industrie [Confederación Española de Organizaciones Empresariales] oppose this agreement. How do you view this agreement?

Summary statistics (Climate change agreement)

Table A-9: Summary statistics (Climate change agreement)

	N	Mean	St. Dev.	Min	Median	Max
Paris agreement approval	3,810	6.17	1.23	1	7	7
Trust Friends of the Earth	3,076	5.98	2.80	1	6	11
Trust big business	3,885	4.71	2.43	1	5	11
Trust business association	3,554	4.35	2.47	1	4	11
Spain	2,001	0.50	0.50	0	1	1

Regression tables

Table A-10: Regression results

	**Model 18**	**Model 19 (Germany)**	**Model 20 (Spain)**
Trust Friends of the Earth	0.148	0.236	0.039
	(0.018)	(0.024)	(0.029)
Trust companies	−0.048	−0.033	−0.064
	(0.023)	(0.030)	(0.035)
Trust bus. assoc.	−0.002	−0.030	0.051
	(0.024)	(0.031)	(0.039)
Friends of the Earth pro	−0.768	−0.483	−0.906
	(0.276)	(0.422)	(0.394)
Friends of the Earth con	−1.643	−0.680	−2.169
	(0.276)	(0.424)	(0.392)
Companies pro	−0.612	−0.587	−0.630
	(0.271)	(0.343)	(0.454)
Companies con	0.439	0.709	0.013
	(0.265)	(0.359)	(0.399)
Bus. assoc. pro	−0.494	−0.225	−0.807
	(0.252)	(0.336)	(0.396)
Bus. assoc. con	0.047	−0.077	0.279
	(0.266)	(0.358)	(0.412)
Spain	0.647		
	(0.086)		
Trust FoE × FoE pro	0.057	0.032	0.049
	(0.041)	(0.058)	(0.072)
Trust FoE × FoE con	0.008	−0.097	0.037
	(0.041)	(0.057)	(0.069)
Trust companies × Companies pro	0.056	0.063	0.049
	(0.050)	(0.061)	(0.088)
Trust companies × Companies con	−0.133	−0.159	−0.088
	(0.048)	(0.063)	(0.075)
Trust bus. assoc. × Bus. assoc. pro	−0.018	−0.046	0.007
	(0.047)	(0.059)	(0.083)
Trust bus. assoc. × Bus. assoc. con	−0.075	−0.041	−0.148
	(0.049)	(0.063)	(0.080)
N	2751	1654	1097
Log Likelihood	-3386.917	-2108.576	-1254.813

Note: Ordinal regression coefficients with standard errors in parentheses
